# Repurposing drugs to target the malaria parasite unfolding protein response

**DOI:** 10.1038/s41598-018-28608-2

**Published:** 2018-07-09

**Authors:** Yun Chen, Claribel Murillo-Solano, Melanie G. Kirkpatrick, Tetyana Antoshchenko, Hee-Won Park, Juan C. Pizarro

**Affiliations:** 1 Department of Molecular Biology and Biochemistry, School of Medicine, Tulane University, New Orleans, USA; 2 Department of Tropical Medicine, School of Public Health and Tropical Medicine, Tulane University, New Orleans, USA; 3 Vector Borne Infectious Disease Research Center (VBIDRC), Tulane University, New Orleans, USA

## Abstract

Drug resistant *Plasmodium falciparum* parasites represent a major obstacle in our efforts to control malaria, a deadly vector borne infectious disease. This situation creates an urgent need to find and validate new drug targets to contain the spread of the disease. Several genes associated with the unfolded protein response (UPR) including Glucose-regulated Protein 78 kDa (GRP78, also known as BiP) have been deemed potential drug targets. We explored the drug target potential of GRP78, a molecular chaperone that is a regulator of the UPR, for the treatment of *P. falciparum* parasite infection. By screening repurposed chaperone inhibitors that are anticancer agents, we showed that GRP78 inhibition is lethal to drug-sensitive and -resistant *P. falciparum* parasite strains *in vitro*. We correlated the antiplasmodial activity of the inhibitors with their ability to bind the malaria chaperone, by characterizing their binding to recombinant parasite GRP78. Furthermore, we determined the crystal structure of the ATP binding domain of *P. falciparum* GRP78 with ADP and identified structural features unique to the parasite. These data suggest that *P. falciparum* GRP78 can be a valid drug target and that its structural differences to human GRP78 emphasize potential to generate parasite specific compounds.

## Introduction

Malaria is a worldwide public health problem with an estimated 600,000 deaths per year^1^. Due to the lack of an efficacious vaccine^2^, prevention and chemotherapy are the two available methods to control the impact of malaria^3^. Current antimalarial drugs target primarily *P. falciparum*, the most important parasite in terms of malaria-associated mortality and morbidity^4^. Unfortunately, drug-resistant *P. falciparum* strains are very common worldwide^5,6^. Thus, there is a constant need to identify and validate new antimalarial drug targets to sustain current disease control strategies. To meet this demand we explore the stress response pathway, which includes multiple chaperones that have been already validated as drug targets in other diseases, and have been suggested as potential new antiplasmodial drug targets^7,8^.

GRP78 is a molecular chaperone that resides in the lumen of the endoplasmic reticulum (ER)^9^. Its function is to bind newly formed polypeptides translocated into the ER and to assist them to reach their native folded state^10^. GRP78 function is essential in maintaining ER homeostasis, and consequently essential for the synthesis, folding and modification of membrane and secreted proteins. Several stress situations (*e.g*., nutrient deprivation, chemical stress) can perturb normal ER functions leading to the accumulation of misfolded proteins that overload the ER capacity. ER stress triggers the unfolded protein response (UPR), an evolutionarily conserved pathway designed to restore normal ER functioning^11^. The UPR coordinates an increase in the ER-folding capacity by up-regulating ER chaperones’ expression and augmenting degradation of misfolded proteins^11^. UPR also diminishes the folding load by attenuating translation and selective degradation of mRNAs^12,13^. Restoring ER homeostasis is cytoprotective since it allows cells to adapt to environmental conditions that impinge on the folding of membrane and secreted proteins^14^. The malaria parasite possesses a functional GRP78^15^, which has been shown to regulate eIF2-α–mediated arrest of protein translation, an essential function during the schizont and gametocyte stages^12,16–18^.

The GRP78 chaperone belongs to the Hsp70 family and it is composed of two domains, a nucleotide-binding domain (NBD) joined by a flexible linker to a substrate-binding domain (SBD). The chaperone function requires ATP hydrolysis, and substrate binding accelerates its ATPase activity^19^. The linkage between chaperone and ATPase activity provides the rationale to use inhibitors that bind to GRP78’s NBD, as pharmacological agents in order to combat diseases that rely on GRP78 function. Previous studies have explored GRP78 inhibitors as potential anticancer therapeutics^20^, validating this approach. Taking advantage of inhibitor availability^21^, we report our work that explored the “druggability” of the *P. falciparum* GRP78 (PfGRP78) chaperone by a combination of x-ray crystallography, protein binding assays and inhibitor testing against *in vitro P. falciparum* cultures. Our structural and biochemical characterization of the parasite protein identified a significant difference in flexibility from the human chaperone, a contrasting feature that could be used to generate *Plasmodium* specific GRP78 inhibitors. Also, we correlated inhibitors’ binding with their anti-parasitic activities *in vitro*, further validating our approach. In conclusion, chemical targeting of the *P. falciparum* GRP78 chaperone appears to be a viable avenue to identify new drug leads against malaria.

## Results

### PfGRP78 ATP binding domain structure

Two distinct recombinant *P. falciparum* GRP78 protein constructs were successfully expressed and purified from bacterial culture. The longest construct dubbed PfGRP78-FL, included the nucleotide and the substrate binding domains, residues S24 to K629. Two mutations were introduced in this construct T226A and 449-TYQDNQP-455 to VGG to mimic the ATP bound state. It has been shown that these changes are essential to express the full-length protein^22^. The second construct encompassed the ATP binding domain (residues I26 to G404) and it will be referred as PfGRP78-NBD. Both protein constructs were used in the crystallization trials. However, only PfGRP78-NBD in complex with ADP produced high-resolution diffracting crystals.

The crystal structure of PfGRP78-NBD in complex with ADP was determined at 2.3 Å resolution, and the final model included four chaperone-nucleotide complexes in the asymmetric unit related by non-crystallographic symmetry (NCS). As expected, all of the molecules showed unambiguous electron density for ADP and a Mg^2+^ atom in the active site, since these compounds were added prior to the crystallization process. However, the presence of an additional PO_4_ group in the active site was surprising. All the molecules in the asymmetric unit shared the same conformation with an average r.m.s.d of 0.3 Å over all atoms (0.15 Å for main chain atoms only). Thus, the following structure description refers to molecule A on the asymmetric unit.

**Figure 1:**
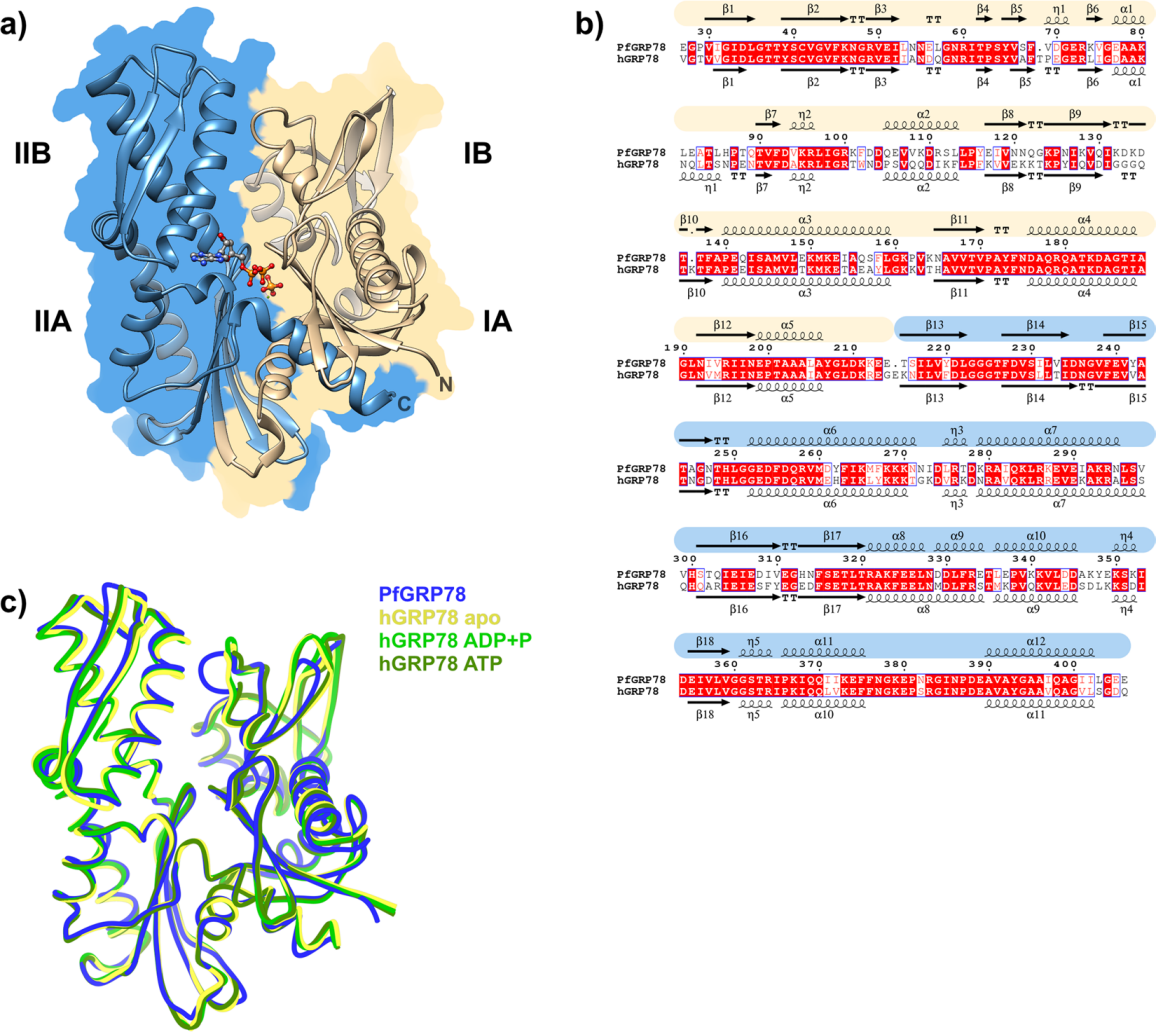
Crystal structure of PfGRP78-NBD and comparison with the human GRP78 structures. (**a**) Cartoon representation of PfGRP78-NDB with each lobe colored differently (I – yellow and II – blue), with ADP and PO_4_ shown in ball and stick representation. (**b**) Sequence comparison between *P. falciparum* and human GRP78 NBDs. Secondary structure is shown above and below its corresponding sequence, and the malaria is color-coded according to the lobe organization as indicated before. (**c**) Structural overlay of malaria and human protein structures.

The PfGRP78-NBD showed a classical HSP70 NBD structure composed by four subdomains (IA, IB, IIA, and IIB) arranged in two lobes, with the ATP binding site located at the bottom of the crevice between lobes I and II (Fig. 1). The N-terminal lobe I included residues 26 to 215 arranged in 12 β-strands and 5 α-helices, while the C-terminal lobe II (residues 216–403) was composed of 6 β-strands and 7 α-helices (Fig. 1b). The lobe interface included 115 residues, 19 interactions and 2210Å^2^ of buried surface area. The interactions between subdomains IA and IIA comprised ~85% of the lobe interface, and the subdomains accounted for 90% of the polar interactions. This domain arrangement created a twisted v-shaped lobe orientation.

**Figure 2:**
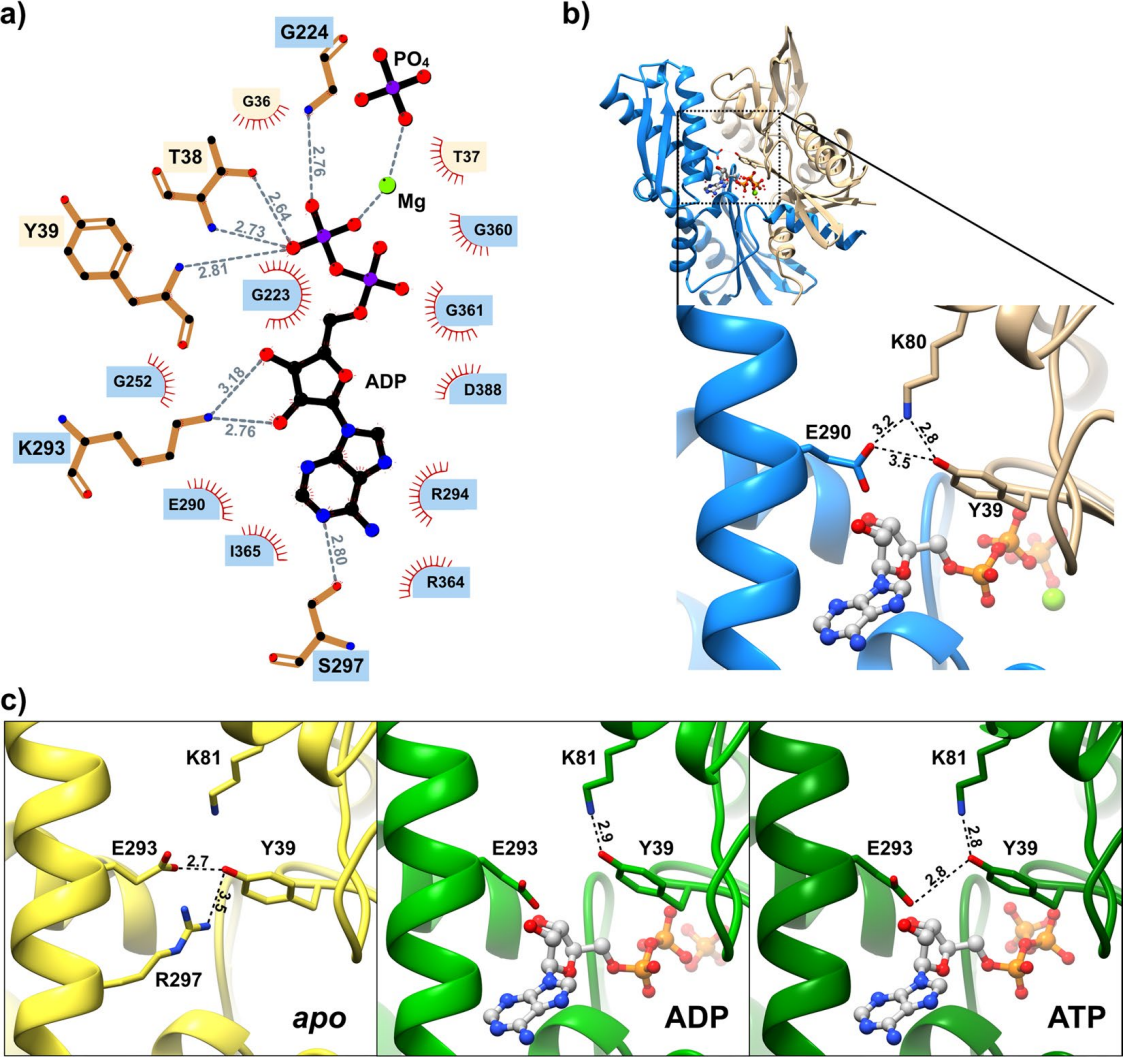
Malaria GRP78–ADP interactions. (**a**) Phosphonucleotide interacting residues of PfGRP78. H-bond interactions are indicated by gray dash lines and distances are reported in Å. Each residue is shaded according to the lobe where it belongs, in the same color code as Fig. 1. (**b**) Detailed view of the Tyr39, K80 and Glu290 interactions in PfGRP78-NBD. (**c**) Detailed view of the Tyr39, K81 and Glu293 interactions in huGRP78-NBD.

In PfGRP78, the ATP binding site was sandwiched between the IA-IIA and IB-IIB subdomain interfaces (Fig. 1a). But, ADP recognition was not symmetrically distributed among them. The bulk of the interactions with the base and sugar portions were associated with the IIA and IIB subdomains, including all the residues involved in H-bond contacts. While the phosphate moiety and the PO_4_ made extensive contacts, including 7 H-bonds, with residues from the IA and IIA subdomains; residues from the IB subdomain provided additional phosphate contacts; this subdomain also created a lid that trapped the ATP hydrolysis products (Fig. 2a). The presence of ADP and PO_4_ in the active site is consistent with a post-hydrolysis state, and it has been previously shown that product release is the rate-limiting step in GRP78 and other HSP70 ATPases^19^.

### Structural comparison between *P. falciparum* and human GRP78 NBDs

The malaria parasite and its human host GRP78 chaperones shared an overall 84.4% sequence similarity that increased to 87.8% when the comparison was restricted to the NBD alone (Fig. 1b). To evaluate their structural differences, we superimposed the PfGRP78-NBD structure with three different human GRP78 NBD structures, *apo* and the complexes with ADP plus PO_4_ and with ATP (PDB id 3LDN, 5EVZ and 5F1X respectively). Overall, both *Plasmodium* and human chaperone ATP binding domains shared the same arrangement of secondary structure elements and their three-dimensional structures were very similar with less than 1 Å r.m.s.d over all atoms.

The presence of ATP in the human chaperone was associated with the highest structural similarity to the parasite structure (Table 1). The largest r.m.s.d. difference was observed when the PfGRP78-NBD was overlaid to the human *apo* structure, whereas the human ATP complex structure showed the lowest difference when compared to the malaria, 0.76 Å on average over main chain atoms. These differences and the fact that nucleotide binding was mostly associated with the C-terminal lobe II, prompted us to superpose each lobe independently (Table 1). All human GRP78 structures regardless of their ligand appeared more similar to the *Plasmodium* structure when only lobe II residues were superposed. The relatively high r.m.s.d. values seen with lobe I superposition were also associated with lower sequence identity between the human and the malaria parasite proteins, 50% compared to 74% between lobe II residues (Fig. 1b). Despite these sequence differences all residues interacting with ADP are conserved between malaria and human GRP78 (Figs 1b and 2a).

**Table 1:** Structural comparison between malaria and human GRP78 NBD crystal structures.

	PfGRP78-NBD (ADP + PO_4_)			
	NBD		Lobe I	Lobe II
	Main chain	All atoms	Main chain	Main chain
hGRP78-NBD (apo)	0.800	0.992	0.806	0.538
hGRP78-NBD (ADP + PO_4_)	0.745	0.981	0.865	0.538
hGRP78-NBD (ATP)	0.696	0.886	0.797	0.555

R.m.s.d. (root-mean-square deviation) values in Å are indicated for the overlay between the malaria protein and several human GRP78 structures, *apo* (PDB id 3LDN) and in complex with ATP (PDB id 5F1X) and ADP (PDB id 5EVZ). R.m.s.d. values for main chain and all atoms comparisons are reported for equivalent residues.

The sequence and structural variation between the two GRP78 lobes were accompanied by changes in the lobe I–lobe II interacting surface. All four molecules of PfGRP78-NBD in the asymmetric unit showed large inter-lobe interacting surfaces involving 117 residues and an average buried surface area of 2230Å^2^ (range from 2168 to 2313Å^2^) (Supplementary Information; Table S1). The human structures were more variable in buried surface area ranging from 2060 to 2321Å^2^, suggesting different lobe I- lobe II orientations. Surprisingly, the buried surface area variation among the human GRP78 structures was not correlated with the presence of a phosphonucleotide in the active site (Table S1). Buried surface areas of the two asymmetric molecules in the human ATP structure differed by 212Å^2^, while in the apo structure the difference between the two NCS related molecules was even larger, 252Å^2^. The ADP complexes, both the human and malaria, showed smaller differences among the NCS related molecules with differences of 73 and 75.8 Å^2^ respectively. But, the PfGRP78-NBD lobe I–lobe II interaction surface was on average 104Å^2^ larger than the area of the human ADP complex. These observations showed that the parasite GRP78 NBD has less variability in the lobe I–II orientation compared to the human protein. Further analysis of the interacting residues of the malaria parasite chaperone showed two H-bonds linking IB and IIB subdomains. The first was between residues Y39 and K80 (K81 in human), and the second included residues K80 and E290 (E293 in human) (Fig. 2b). These interactions were present in all four molecules of the asymmetric unit in the PfGRP78-NBD crystal structure, but were absent or not always present in the human protein. While the K81-E293 H-bond was absent in all human structures, the K81–Y39 interaction was present only in human structures that included a bound nucleotide (Fig. 2c). An additional H-bond between Y39 OH…Oε_2_ E293 was observed in hGRP78-NDB *apo* structure in the molecule with the largest inter-lobe interfaces over 2300Å^2^. However, in PfGRP78-NBD the angle of the Y39-E290 interaction was too acute (~10°) to be classified as an H-bond interaction (Fig. 2c and Table S1).

To evaluate the role of the Y39, K80 and E290 interaction in PfGRP78-NBD conformational stability and its impact in ligand binding, a mutant protein was generated to eliminate the H-bond donor by replacing the tyrosine with a phenylalanine (Y39F). The observation of a direct H-bond in hGRP78-NBD structures with large lobe I and II surface of interaction was our main rationale to mutate Y39. We assumed that the Y39-E290 H-bond is present in PfGRP78-NBD in the absence of a nucleotide as seen in the *apo* hGRP78-NDB structure (Fig. 2c), and that ATP binding disrupts this interaction. This assumption is supported by the observation that nucleotide binding leads to a rotation of the lobe II with respect to the lobe I to render accessible the binding site at the lobe I and II interface^22^. The PfGRP78-NBD mutant chaperone was significantly less stable than the native protein, as evidenced by a lower melting temperature (*T*_*m*_), wt = 41.2 °C vs. Y39F = 37.8 °C. The thermal stability experiment suggests that the Y39F mutation disrupts an important interlobe hydrogen bond, and the absence of the interaction was the cause of destabilization. Thus, the consistent presence of the Y39-K80-E290 interaction in PfGRP78-NBD, and the inconsistent presence of the same interaction in human chaperone, makes a small but significant contribution to the malaria parasite protein rigidity.

### GRP78 NBD phosphonucleotide and inhibitor binding

**Figure 3:**
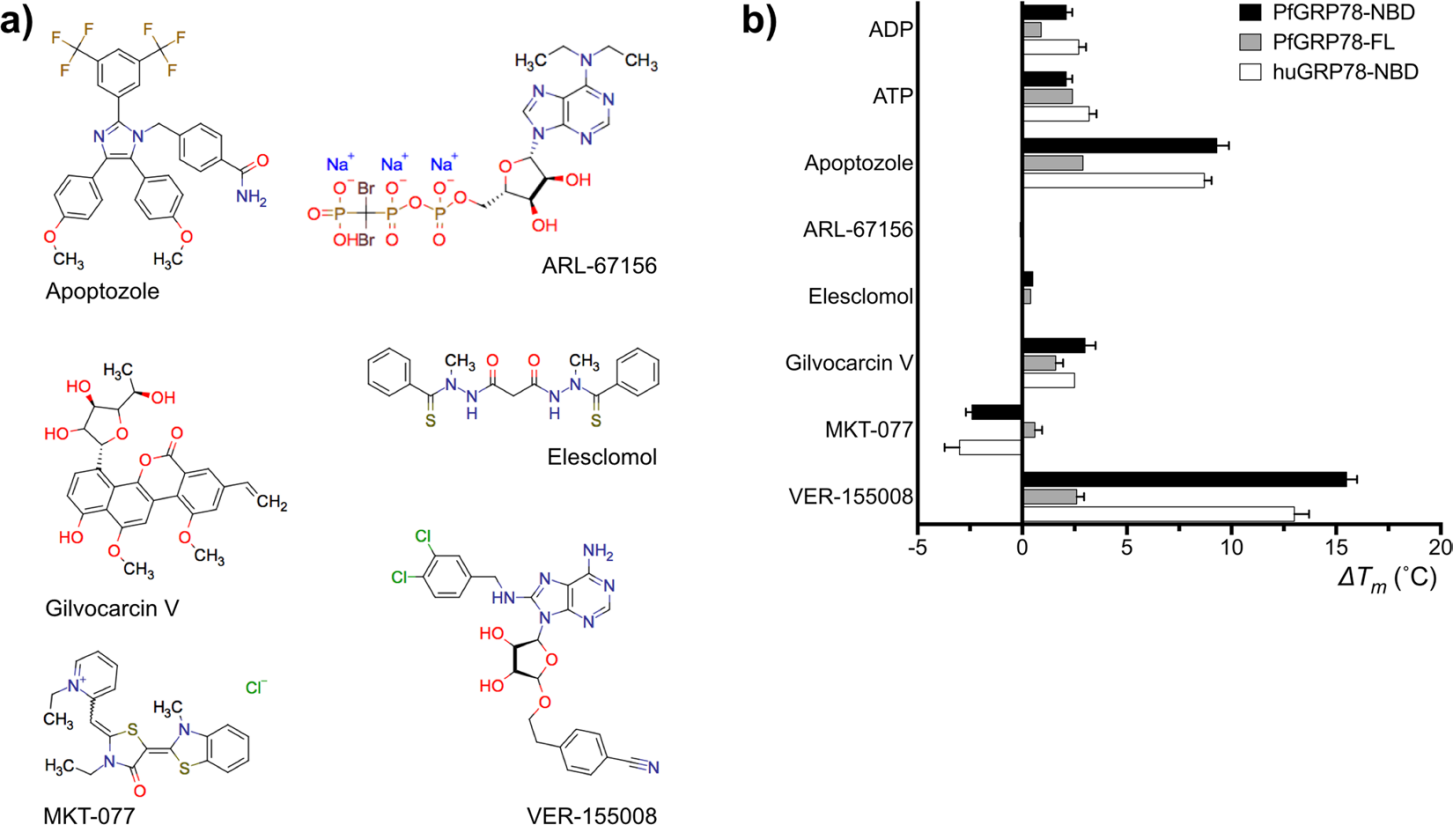
Interactions of GRP78 inhibitors with malaria and human GRP78. (**a**) Chemical diagrams of the six inhibitors tested. (**b**) DSF results for malaria (full-length and NBD) and human (NBD) GRP78 interaction with selected inhibitors. ΔΔ*C*_*t*_ (°C) values represent an average of three measurements and the standard deviation is indicated.

The *Plasmodium* and human GRP78 NBD differences were further characterized by measuring their interaction with ATP, ADP, and several compounds referenced in the literature as GRP78 inhibitors (Fig. 3a). Initially, their binding was determined using a DSF (Differential Scanning Fluorimetry) assay. In this assay, an interaction between the chaperone and a compound was measured as a change in the protein’s melting temperature (*T*_*m*_), by subtracting the *T*_*m*_ of the protein by itself to the melting value of the protein in the presence of the compound. Differences above or below 1 °C between the melting temperatures (Δ*T*_*m*_) were considered indicative of a protein-compound interaction. As expected, the *Plasmodium* GRP78 chaperone interacted with both ATP and ADP. Four of the six compounds tested had significant effects on protein thermal stability (Fig. 3b).

There were disparities in the Δ*T*_*m*_ values when comparing PfGRP78 FL vs. NBD. While both proteins showed a similar binding profile, the absolute values were quite different. This variation was expected since the two proteins had different thermal stability, and previous work has clearly shown that individual domains behave different than full-length proteins in the DSF assay^23^. Nonetheless, two interactions diverged between PfGRP78-FL and PfGRP78-NBD outside of the expected range. First, the mutated PfGRP78-FL construct showed limited binding to ADP; it was a predictable result because the mutations introduced favored the ATP-bound conformation and prevented the structural changes associated with ATP hydrolysis and product release. Second, both the *Plasmodium* and human GRP78-NBD were destabilized by MKT-077, while the modified full-length protein showed no evidence of interaction (Δ*T*_*m*_ = 0.6 °C). This finding suggested that the MKT-077 binding site may be occluded or sterically hindered in the full-length protein.

The MKT-077 compound was one of the four compounds that interacted with PfGRP78-NBD according to the DSF assay. As previously noted this interaction led to a significant thermal destabilization of the protein. The other three compounds (apoptozole, Gilvocarcin V and VER-155008), however, increased the thermal stability of PfGRP78-NBD, suggesting the formation of a protein-compound complex. Finally, two compounds (ARL-67156 and Elesclomol) were classified as non-binders based on the DSF assay’s no shift results.

To quantify the binding affinities of PfGRP78-NBD for the selected compounds, dissociation constants (*K*_*d*_) were determined by surface plasmon resonance (SPR). As mentioned above, the results showed a large difference in the ADP and ATP binding affinities between *Plasmodium* and human GRP78 NBD proteins (Table 2). The malaria chaperone showed a 33- and 23-fold decrease in affinity towards ADP and ATP, compared to the human counterpart. This result was consistent with the idea that a more rigid inter-lobe association was partially responsible for the lower affinity of the parasite protein towards the phosphonucleotides. PfGRP78-NBD Y39F mutant showed a twofold increase in affinity towards ADP compared to wildtype, further supporting the notion that protein rigidity affected nucleotide binding. The malaria parasite chaperone also showed low affinity for the inhibitor VER-155008, but not an statistically significant difference (*p* = 0.06). *Plasmodium* and human GRP78, however, showed similar affinities for Apoptozole and MKT-077. Gilvocarcin A binding to human and malaria GRP78 was too weak to accurately determine its dissociation constant by SPR. Finally, there was an agreement between the results from our two independent binding assays, SPR and DSF. The two non-binder compounds (ARL-67156 and Elesclomol) identified by DSF also failed to show any binding in SPR. Finally, PfGRP78-NBD Y39F failed to bind Apoptozole in the two assays, which suggested that the mutation significantly affected the compound-binding site.

**Table 2:** Nucleotide and inhibitor affinity of malaria and human GRP78.

	PfGRP78-NBD wt	PfGRP78-NBD mut	huGRP78-NBD
ADP*	16.2 ± 2.8	8.11 ± 0.73	0.49 ± 0.02
ATP*	333 ± 74	312 ± 15	14.0 ± 7.0
Apoptozole	4.27 ± 0.86	N.B.	6.9 ± 0.33
MKT-077	16.4 ± 1.7	—	16 ± 0.17
VER-155008	164 ± 13.6	—	52.7 ± 27
Gilvocarcin V	N.B.	—	N.B.
ARL-67156	N.B.	—	N.B.
Elesclomol	N.B.	—	N.B.

The affinity constants (*K*_*d*_) determined by SPR are indicated in µM plus/minus their standard error (SE). Statistically significant differences between the two PfGRP78 and the host huGRP78 by an * sign (*p* < 0.05). N.B. = No binding; “—“ = Not determined.

### In vitro antiplasmodial activity of GRP78 inhibitors

A major goal of our study was to evaluate the drug target potential of *P. falciparum* GRP78, thus we evaluated the effect of the chaperone inhibition on the malaria parasite. Our approach took advantage of several GRP78 inhibitors (Fig. 3a) previously characterized against human cancer cell lines, which were commercially available^20,24–27^. These compounds were tested against two different parasite strains with opposite sensitivity towards the antimalarial drug chloroquine, 3D7 (sensitive) and W2 (resistant). *In vitro* cultures of these malaria parasites were exposed to a concentration series of each compound in a growth inhibition assay to determine its half maximal effective concentration (EC_50_) (Table 3). Three out of the five compounds tested showed strong antiplasmodial activity with MKT-077, Gilvocarcin V and Elesclomol having submicromolar EC_50_ values, which were in the same range as the antimalarial drug chloroquine. The two remaining compounds, Apoptozole and VER-155008, were less toxic against the malaria parasite with EC_50_s in the micromolar range. Only Apoptozole showed a statistically significant activity between the chloroquine sensitive and resistant *P. falciparum* strains.

**Table 3:** *In vitro* growth inhibitory activity of selected GRP78 inhibitors against two *P. falciparum* strains (3D7 and W2) and a human cell line (HCT-116).

	Plasmodium falciparum		Human
	3D7	W2	HCT-116
CQ**	0.05 ± 0.011	0.23 ± 0.03	N.D.
DHA**	0.0004 ± 0.00007	0.0001 ± 0.00002	N.D.
MKT-077^##^	0.07 ± 0.02	0.07 ± 0.03	0.98 ± 0.23
Apoptozole*	6.8 ± 1.4	4.5 ± 0.4	16.46 ± 12.77
VER-155008^#^	82.1 ± 49.7	39.5 ± 0	15.71 ± 0.07
Gilvocarcin V	0.02 ± 0.004	0.08 ± 0.04	N.D.
Elesclomol	0.05 ± 0.017	0.06 ± 0.01	N.D.

Half effective concentrations are reported, EC_50_(µM). Statistically significant differences between the two *Plasmodium* strains are indicated by an *, and between them and the host cells by a # sign. Each value represents the average of three independent measurements plus/minus the standard deviation. N.D. = Not determined. Statistical significance, *p < 0.01, **p < 0.001, ^#^*p* < 0.05 and ^##^*p* < 0.001.

Three out of the five tested compounds against *P. falciparum* strains *in vitro* showed also binding to PfGRP78-NBD. However, there was a very poor correlation between EC_50_ and *K*_*d*_ values for, MKT-077 and VER-155008. The discrepancies between the binding and the antiplasmodial activity showed stronger than expected activity for MKT-077 and VER-155008. Nonetheless, the EC_50_ value recorded for Apoptozole was clearly in line with its *K*_*d*_. Finally, these three GRP78 inhibitors showed different growth inhibition effects on host cells, but the difference was statistically significant only for MKT-077 and VER-155008 (Table 3). VER-155008 selectivity index was 3.8 in favor of host cells, but Apoptozole and MKT-077 were 2.9 and 14 times more selective towards *P. falciparum*.

**Figure 4:**
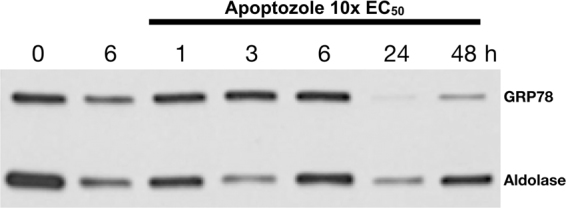
*In vitro* effect of Apoptozole on PfGRP78 protein levels. A time course of PfGRP78 protein levels in *P. falciparum* W2 cultures exposed to 10xEC_50_ (45 µM) Apoptozole (lanes 3 to 7) or untreated control (Lane 2). Western-blot revealed with a polyclonal anti-PfGRP78 (courtesy of Dr. N. Kumar, Tulane University) and an anti-PfAldolase serving as a loading control. The whole western-blot scan is shown as Supplementary Information (Fig. S1).

The effect of Apoptozole in PfGRP78 was further evaluated *in vitro* by exposing a *P. falciparum* W2 culture to a concentration of the inhibitor equal to 10 times its EC_50_ (45 µM) for 6 h. Culture aliquots were taken at several time points and the PfGRP78 levels of were evaluated by immuno-blots (Fig. 4). Upon exposure to Apoptozole the chaperone levels did not changed during the first 6 hours, but at the 24 and 48 h time points there was a clear reduction in the PfGRP78 levels. This observation suggested that the Apoptozole GRP78 interaction lead to the degradation of the chaperone. Thus, providing further support to the notion that Apoptozole antiplasmodial activity is mediated by its effect on PfGRP78.

## Discussion

We have sought to validate *P. falciparum* GRP78 as a potential drug target against malaria. The chaperone is an attractive drug target due to its role coordinating the UPR, an important component of the malaria parasite stress response^8^. Significantly, this pathway and GRP78 in particular have been shown to be involved in *P. falciparum* resistance against artemisinin^28^, the main component of drug combination treatments against malaria worldwide. To validate PfGRP78 as a potential drug target we undertook a two-pronged approach. On one side, we characterized inhibitors, reported in the literature as effective against the human chaperone, for their ability to engage the malaria chaperone and their anti-parasitic activity *in vitro*. On the other side, we determined the crystal structure of PfGRP78-NBD to rationalize the binding data and identify *Plasmodium*’s unique structural features with respect to its human counterpart.

Six commercially available GRP78 inhibitors were used in our studies (Fig. 3a), and the results from two biophysical assays clearly showed that four of them bind to the recombinant *Plasmodium* and human GRP78-NBD proteins. The binding results are in agreement with reported data from these compounds. The non-binder compounds, ARL-67156 and Elesclomol^25^, have not been previously tested for direct binding against the protein target, but predicted to inhibit GRP78 based on cell data. In contrast, all the binder compounds Gilvocarcin V, Apoptozole, MKT077 and VER-155008 have been reported to directly interact with GRP78 or other Hsp70 family member. Gilvocarcin V mechanism of action was associated with GRP78 by cross-linking^26^, and Apoptozole^29^, MKT-077^27^ and VER-155008^30^ have been previously characterized for binding to recombinant GRP78. As expected, all these four compounds also engaged the *Plasmodium* chaperone in our two binding assays. The broad specificity of the GRP78 inhibitors towards multiple members of the HSP70 family might help explain the cross-species activity of these compounds.

The compounds’ lack of target selectivity between GRP78 and other members of the Hsp70 family might have limited the drug development efforts against the ER chaperone in the anticancer field^21^. However, for antimalarial drugs the lack of selectivity will be a liability only if it is associated with significant host cytotoxicity. In our case, higher affinity towards the host chaperone was correlated with increased host cell toxicity. VER-155008 was on average eight times more selective to host cells, and its affinity towards hGRP78-NDB was three times lower compared to PfGRP78-NDB. However, Apoptozole and MKT-077 that showed similar binding affinities between the malaria and host chaperones were more selective towards the parasite in the growth inhibition assay, 2.5 and 13 times respectively. This suggests that the parasite survival is more sensitive to disruption of the chaperone function than its host, as it has been previously suggested for the Hsp90 chaperone^31^.

Despite the limited selectivity of GRP78 inhibitors, VER-155008 showed a difference in affinity between the parasite and human proteins (Table 2). The *Plasmodium* chaperone’s lower affinity for the inhibitor followed the same trend as its limited affinity towards the phosphonucleotides. In the case of ADP and ATP, however, the differences between the malaria parasite and human GRP78-NDBs were more pronounced, 33 and 23-fold reduction in affinity respectively. We propose that the lower substrate, product and inhibitor binding affinities observed for PfGRP78-NBD compared to the human chaperone are a consequence of a more rigid lobe I-II interface in the malaria protein. The PfGRP78-NBD lobe interface is on average 56Å^2^ larger and contains additional H-bonds when compared with its human counterpart. The fact that affinity differences are limited to VER-155008, an ATP competitive inhibitor known to bind to the nucleotide binding site, further support our rigidity hypothesis. This is because other GRP78 inhibitors, such as Apoptozole^29^ and MKT-077^32^, predicted to bind elsewhere in the protein showed no significant differences in affinity between the host and parasitic proteins.

Our results suggest that the binding affinity differences between the malaria and human chaperones are related to the overall rigidity of the nucleotide-binding domain. The position of the nucleotide-binding site at the base of the v-shaped structure makes substrate binding dependent on the rotation of one lobe with respect to the other. This is an essential feature of GRP78 and other Hsp70s that allows the allosteric coupling of ATPase and chaperone activities^33^. In the PfGRP78-NBD crystal structure, the interface area between lobes I and II showed less variability compared with human structures, which suggests more rigid protein in the malaria parasite. This rigidity was accompanied with a decrease in affinity for the phosphonucleotides when compared to the human chaperone. The reduction in affinity could result from the need for additional energy necessary to rotate one of the lobes that allow access to the nucleotide-binding site. A major difference between *Plasmodium* and human GRP78 was the presence of two H-bonds between residues Y39-K80-E290 associated with subdomains IB and IIB interaction. The mutagenesis results highlighted the contribution of these H-bonds to the overall stability of the malaria protein. The mutant Y39F showed a less thermal stable NBD accompanied with a concomitant increase in ADP and ATP affinity, suggesting that a weaker surface interaction between the subdomains facilitates the subdomain rotation necessary for the binding of the nucleotides. The impact of target flexibility in the drug discovery process has been recognized for more than twenty years^34^, and differential protein dynamics have been validated as an approach to identify and develop target specific inhibitors^35^.

Knowledge of the crystal structure of a target enables the use of structure-based drug design methods. Our comparative structural analysis of *P. falciparum* and human GRP78-NBDs highlighted the increased structural rigidity in the parasite protein, which impacts inhibitor design in two ways. On one hand, the rigid PfGRP78 would be a disadvantage for inhibitor discovery because potential hits need to have structural complementarity to the nucleotide binding site with limited structural rearrangements. On the other hand, the rigid PfGRP78 is better suited for computational screen of bioactive compounds, since active site flexibility is computationally expensive. Moreover, the poor affinity of PfGRP78 for the endogenous ligands, ADP and ATP, compared to the human chaperone, provides an additional advantage for chemical inhibition. In general, the majority of hits bind with relatively low affinity and compound potency will be negatively affected by the high ADP/ATP affinities of a target protein. Low affinities of PfGRP78 for the phosphonucleotides will work to one’s advantage during the drug discovery process because this favors competitive inhibition.

Three compounds showed distinct growth inhibitory effects against the malaria parasite *in vitro*. MKT-077 was the compound with the strongest *in vitro* growth inhibition activity against both strains of *P*. *falciparum*, followed by the Apoptozole and VER-155008. But, a match between the compound’s EC_50_ and its *K*_*d*_ was observed for only one inhibitor. On the one hand, MKT-077 and VER-155008 anti-plasmodial effects were one to two orders of magnitude better than their *K*_*d*_ values. Undetermined off-target effects of these compounds could explain the discrepancies between target affinity and anti-parasitic activity. On the other hand, Apoptozole’s affinity constant and EC_50_ data were both in the 1–10 µM range. In addition, malaria parasites exposed to an Apoptozole concentration ten-times its EC_50_ showed a reduction of PfGRP78 levels. We suggest that the binding of Apoptozole to PfGRP78 leads to the degradation of the chaperone, negatively impacting its protein substrates. This suggested mode of action is similar to other chaperone inhibitors^36^. Our combined observations support the conclusion that Apoptozole’s effect on PfGRP78 is responsible for its anti-parasitic activity. The suggested mode of action for Apopotozole is consistent with an idea that the sole inhibition of GRP78 by a compound can be lethal for the parasite, thereby reinforcing PfGRP78 as a potential drug target to combat malaria.

In conclusion, our results established PfGRP78 as a potential drug target against *P. falciparum*, and identified Apoptozole, a novel chemical scaffold that inhibits chaperone function and prevents parasite growth *in vitro*. Apopotozole showed an EC_50_ in the micromolar range, and a similar binding affinity towards the recombinant chaperone. This compound binding is affected by the Y39 mutation, thus suggesting it binds to the interface between lobe I and II near the ATP binding site that showed significant structural differences between the *Plasmodium* and human proteins. Future structural information of the PfGRP78-NBD–Apoptozole interaction can guide the development of parasite specific inhibitors. Finally, the identification of Apoptozole as a PfGRP78 inhibitor open the possibility to use this compound as a chemical probe to study the role if this chaperone in artemisinin resistance mechanisms.

## Methods

### Growth Inhibition Assay

A synchronized *P. falciparum* culture, with over 80% ring forms, was used in the growth inhibition assay. Each strain was assayed at 0.5% parasitaemia and 1.5% hematocrit. A 190 µL aliquot of parasite culture was dispensed per well to microplates that were pre-dispensed with the compounds at various dilutions. A 10 µL aliquot of 11 dilutions per compound had been previously dispensed into a 96-well flat-bottom, in duplicate. Dilutions of GRP78 inhibitors and DHA were prepared in water with a final concentration of DMSO below 0.05% (v/v). Our preliminary assays determined that this DMSO concentration did not impact *P. falciparum* growth when compared to non-exposed cultures (data not shown). Control parasites and parasites exposed to CQ dilutions did not contain any DMSO. The drug/inhibitor dilutions cover the following ranges: CQ from 3.12 nM to 3.2 µM; DHA from 0.05 to 128 nM; Apoptozole from 260 nM to 15 µM, Gilvocarcin V from 4.88 nM to 5 µM, VER-15008 from 0.488 µM to 500 µM, Elesclomol and MKT-077 from 0.488 nM to 500 nM. The microplates were incubated at 37 °C in a controlled atmosphere (5% O_**2**,_ 5% CO_2_ Nitrogen balanced) for 72 hours. After incubation, the plates were subject to a freeze-thaw cycle (−80 °C) and the parasite growth was quantified by the SYBR Green I method as previously described^37^. Briefly, 100 µl of homogenized parasite culture was transferred into 96-well black plates. 100 µl of SYBR Green lysis buffer (2x SYBR Green I, 100 mM Tris-HCl pH 7.5, 10 mM EDTA, 0.016% Saponin and 1.6% Triton X-100) were added to each well. The plates were incubated at room temperature in darkness for 1 hour. The fluorescence signal was read in a Synergy HT (Biotek Instruments, Inc., Winooski, VT) plate reader with 485_Ex_/520_Em_ nm.

The 50% inhibitory concentrations (IC_50_) for each compound were calculated using the online program ICEstimator 1.2 (http://www.antimalarial-icestimator.net/)^38^. IC_50_ values were used in the analysis only if the parasite growth index, determined by the ratio of fluorescence units (FU) between the parasite grown without compound versus signal at maximum compound concentration, was equal to or higher than 2. Growth inhibition experiments were conducted at least three times per compound. The EC_50_ differences for each inhibitor between the two *P. falciparum* strains and human HCT-112 were compared with a *t-*test corrected for multiple comparisons. The statistical analysis was performed with the software Prism 7 (GraphPad Software, San Diego, CA).

### Cloning of P. falciparum GRP78 and protein expression plasmids

The malaria GRP78 (PlasmoDB ID PF3D7_0917900) was cloned from *P. falciparum* 3D7 cDNA. The ATPase domain named PfGRP78-NBD was obtained by inserting a PCR-generated DNA fragment into vector pET28-MHL (*GenBank* accession EF456735) that included residues I26 to A404. The Y39F mutant PfGRP78-NBD was generated by site-directed mutagenesis of the wild-type construct. A PCR was performed using the primers that contained the mutant codon (forward –TTGTATTTCCAGGGCATTGAGGGACCCGTTATTGGTATTGACTTGGGTACCACTTTTAGTTGCGTTGGTG; and reverse - CAAGCTTCGTCATCAACCTAAAATAATACCTGCTTGGATAGCAGCACCATAAGCAACAGCTTCATCAGG) (Integrated DNA Technologies, USA).

The modified full-length PfGRP78 (residues S24 – K629) sequence was commercially synthesized (Life Technologies Corporation, Thermo Fisher Scientific). This construct contains two modifications T226A and _449_TYQDNQP_455_ to VGG) to prevent protein aggregation and ATP hydrolysis, as previously described for the human GRP78^22^. All the protein expressing plasmids were sequence validated.

### Recombinant protein production and purification

PfGRP78 expression vectors were transformed into BL21(DE3) competent *E. coli*, which were used to start a 5 mL LB 50 μg/ml kanamycin culture. After shaking at 220 RPM for 4 hours at 37 °C, 4.5 ml were inoculated into 100 ml LB flasks and incubated overnight at 37 °C. The 100 ml LB culture was transferred into 1 L TB cultures and incubated until OD_600_ reached between 2.0 and 3.0. Protein production was induced with 200 μM IPTG and cell cultures were incubated overnight at 16 °C. Cells were harvested by centrifuging at 3,600 RPM for 20 min at 4 °C, and cell pellets were frozen at −80 °C until purification.

Both PfGRP78 proteins were purified using the same protocol. A frozen cell pellet was thawed and re-suspended in 150 ml of a buffer containing 20 mM Tris-HCl pH 7.5, 200 mM NaCl, 1 mM 2-βME (Buffer A) with additional 0.1% of IGEPAL, 1 mM benzamidine, and 1 mM PMSF. Resuspended cells were disrupted by French Press under a high pressure (22000 psi). The lysate was centrifuged at 15000 RPM for 20 min at 4 °C and the supernatant loaded onto superloop of the fast protein liquid chromatography (FPLC) system for a tandem chromatographic purification, metal affinity and size exclusion.

The sample was completely loaded onto a Nickel column (NiNTA Quiagen, USA), followed by two wash steps using 2% and 8% of buffer B, which contained buffer A plus 250 mM imidazole. An initial elution pool was obtained with 20% buffer B, and the second pool was eluted with 100% buffer B. Both elution pools were collected and named “20W” and “100E”. After metal affinity chromatography, the 20 W and 100E pools were individually loaded onto a size exclusion column, HiLoad 26/600 Superdex 200 pg (GE Healthcare Life Sciences, USA). Fractions (5 ml each) were collected based on UV signal. The purified protein fractions were evaluated by SDS-Page electrophoresis and pooled accordingly. Protein concentration was determined by A_280_ spectrophotometric read (Nanodrop 2000, Thermo Fisher Scientific, USA). The N-terminal His-tag was cleaved with TEV proteases using a ratio of 1:50 for a 4 °C overnight incubation, followed by the additional Nickel column step to remove uncut proteins. For co-crystallization, aliquots of TEV-cleaved PfGRP78-NBD were mixed with 10 × fold molar excess of ADP and Mg^2+^ and incubated overnight at 4 °C. The protein–nucleotide complex was concentrated to ~30 mg/ml.

### Differential scanning fluorimetry (DSF)

DSF screening was carried out using a CFX96 Touch Real-Time PCR Detection System (BioRad, California). The PfGRP78-FL, PfGRP78-NBD wild type and mutant, and human GRP78-NBD were buffered in 25 mM hepes pH 7.5, 250 mM NaCl and assayed in a 96-well format. The final concentration of the protein sample was optimized between 0.05 and 0.2 mg/ml for each protein. The concentration was optimized for each protein to avoid saturation of the fluorescence detector. The GRP78 inhibitors were used at a final concentration of 0.5 mM. SYPRO Orange (Life Technologies, USA) was added as a fluorescence probe in a dilution of 1:1000. The experiments were conducted between 18 °C to 90 °C at a heating rate of 1 °C per minute. The recorded fluorescence reads were fitted to the Boltzmann sigmoid function using DMAN software^39^. The inflection point of each fitted curve is defined as the melting temperature (*T*_*m*_). The observed temperature shift, *ΔT*_*m*_, was recorded as the difference between *T*_*m*_ of the protein with ligand minus *T*_*m*_ of the protein without ligand. Thermal shifts above or below 1 °C were considered significant.

### Biacore Analysis

SPR measurements were performed on Biacore T200 instrument (GE Healthcare) at 25 °C. Purified *Pf*GRP78-FL, PfGRP78-NBD wild-type and mutant, and human GRP78-NBD were immobilized on a CM5 sensorchip using NHS/EDC coupling following the manufacturer protocol to a level of <10,000 RUs, a reference surfaces without immobilized proteins served as a control for nonspecific binding and refractive index changes. Seven different concentrations of the ligands between 0.4 nM to 1 mM, were injected in triplicate over the sensor chip at 30 µL/min in random order. The running buffer was 10 mM HEPES, pH 7.4, 150 mM NaCl, 0.005% P20, 1% DMSO and 2 mM MgCl_2_. Buffer only injections were used as blanks. The Biacore responses recorded during the 30 seconds of the injection were used to estimate the association constant (*k*_*on*_) and the 300 seconds after the injection were used to estimate the dissociation constant (*k*_*off*_). All dissociation and kinetic constants were estimated using double subtracted sensorgrams^40^ using BiaEvaluation Software (GE Healthcare). All the reported parasite dissociation constants (*K*_*d*_) were estimated with affinity analysis, and all the reported human *K*_*d*_s were calculated from kinetic constants. Pairwise affinity differences were evaluated with a *t* test using Holm-Sidak correction for multiple comparisons (alpha = 0.05).

### Determination of in vitro levels of PfGPR78 after Apoptozole exposure

A 55 ml suspension of ring-stage *P. falciparum* W2 parasites at 1.5% parasitaemia and 1.5% of hematocrit were split into 11 culture dishes of 5 ml each. One culture plate was served as a starting point (0 h) and of the remaining ten culture plates, half were exposed to 45 µM Apoptozole and half to DMSO 0.09% for 6 h. After 6 h the Apoptozole or DMSO were removed by washing the parasite culture with complete media twice. Culture dishes contained control (DMSO) and Apoptozole treated parasites were incubated at 37 °C in 5% O_**2**_ and 5% CO_2_ atmosphere throughout the duration of the experiment. One of each control and treated group were harvest at times 1, 3, 6, 24 and 48 hours. Samples were processed by harvesting the red blood cell using centrifugation 5 min at 5000 g and washed twice with PBS 1 × 5000 g for 1 min at 4 °C. The pellet was resuspended in 1 ml of 0.02% saponin-PBS solution (Sigma, Aldrich) and centrifuged 10 min 11000 g at 4 °C. The parasite pellet was washed twice with PBS and resuspended in 100 µl PBS. A 30 µl sample was mixed with 30 µl of Laemlii lysis buffer (BioRad) and 3 µl 14.1 M 2-mercaptoethanol (BioRad) and boiled for 10 min. 10 µl of sample from each time point were separated by SDS-PAGE 4–20% gradient gel (BioRad) and then proteins were transferred to a PVDF membrane. The membrane was blocked overnight with nonfat dry milk 3% in TBS-T. The membrane was incubated with two primary antibodies, a rabbit polyclonal anti-*Pf*GRP78^41^ (kindly provided by Dr. Kumar, Dept. Tropical Medicine, School of Public Health and Tropical Medicine, Tulane University) and a purchased anti-*P. falciparum* aldolase (Abcam); and a secondary donkey anti-rabbit HRP-labeled antibody. The blot was developed using a chemiluminescent substrate (Pierce ECL Western Blotting Substrate) and the signal capture with autoradiography film (Genesee Scientific).

### Crystallization, Data Collection and Structure Solution

Crystallization trials were conducted using the sitting-drop, vapor diffusion method and in-house screening kits, with each drop containing a 1:1 ratio of the complex of PfGRP78-NBD with ADP and Mg^2+^ and the reservoir solution. Drops were set in 96-well Intelliplates (Art Robbins Instruments, USA) and left at a room temperature until crystal growth was observed after two months. Initial crystal hit conditions were optimized using 24-well plates and 1:1 hanging drops. Quality diffracting crystals were obtained after 3 days for the complex by mixing 55.2 mg/ml of the protein with the reservoir solution containing 30% PEG8000, 0.2 M ammonium sulphate and 0.1 M sodium cacodylate at pH 6.5. The crystals were cryoprotected by an equal (1:1) mixture of paratone-N and mineral oil and flash-frozen in liquid nitrogen until data collection.

A 2.3 Å resolution data set was collected at the Canadian Light Source 08ID-1 beamline (Saskatoon, Canada). X-ray diffraction data was processed using the programs XDS^42^, Pointless, and SCALA^43^. The structure was solved by molecular replacement via the program MOLREP in the CCP4 crystallographic suite^43^ using the human GRP78-NBD structure (PDB ID: 5EVZ) after deleting the ligands as a search model processed by Chainsaw. After molecular replacement, the model was built using COOT^44^ and refined using Refmac5^45^ and BUSTER^46^. Summary of data collection and refinement statistics can be found in Table 4. The molecular visualization program Chimera^47^ was used to generate all structural figures.

**Table 4:** Data collection and refinement statistics.

Protein	PfGRP78-NBD
Ligand (actual)	ADP + Mg
PDB id	5UMB
**DATA COLLECTION**	
Beamline	08ID-1 Canadian Light Source Inc. (CLS)
Wavelength (Å)	0.97949
Space group	P 1 2_1_ 1
No. of molecules in asymmetric unit	4
Unit cell parameters (Å, °)	*a* = 84.53, *b* = 112.26, *c* = 93.16 and *β* = 90.13°
Resolution (Å)	48.08–2.30 (2.44–2.30)
No. of measured reflections	259355 (41781)
No. of unique reflections	76738 (12297)
Completeness (%)	99.5 (99.2)
Wilson B factor (Å^2^)	33.03
Friedel redundancy	3.38 (3.40)
R_merge_ (%)	14.0 (62.6)
Average I/σI	7.19 (2.13)
**REFINEMENT**	
Resolution (Å)	48.08–2.30
R_work_/R_free_ (%)	22.7/26.5
No. of atoms	
Protein	11815
Ligand/ion	132
Water	490
Ligand RSCC (%)	96.3
**Average B factors (Å** ^**2**^ **)**	
Protein	33.42
Ligand/ion	23.34
Water	26.22
RMSD bond length (Å)	0.016
RMSD bond angle (°)	1.9°
**Ramachandran analysis**	
Favored (%)	98
Outliers [number] (%)	0 [3]

## Electronic supplementary material

Supplementary Information
