# Supplementary material for: Repurposing drugs to target the malaria parasite unfolding protein response
Source: Sci Rep. 2018 Jul 9;8:10333. doi: 10.1038/s41598-018-28608-2 (PMC6037779; doi:10.1038/s41598-018-28608-2)

**Title**:

Repurposing drugs to target the malaria parasite unfolding protein response

**Authors**:

Yun Chen, Claribel Murillo-Solano, Melanie G. Kirkpatrick, Tetyana Antoshchenko, Hee-Won Park and Juan C. Pizarro.

**Table S1**. Structural characterization of the inter-lobe interface in GRP78 NBD crystallographic structures. Buried surface area, number and nature of inter-lobe interactions are shown. The interatomic distance between the Tyr39 OH, Lys80 N_ζ_ and Glu290 Oε is given (PfGRP78 numbering).

|  | Chain | Buried surface area of interaction (Å^2^) | H-bonds / Salt bridges | Distance  K80 N_ζ_-Oε_2_E290 (Å) | Distance  Y39 OH-N_ζ_ K80 (Å) | Distance  Y39 OH-Oε_2_E290 (Å) |
| --- | --- | --- | --- | --- | --- | --- |
| PfGRP78-NBD – ADP-PO_4_ | A | 2313 | 15 / 3 | 3.29 | 2.98 | 3.47 |
|  | B | 2210 | 17 / 4 | 3.28 | 2.80 | 3.38 |
|  | C | 2230 | 19 / 6 | 3.51 | 2.84 | 3.46 |
|  | D | 2168 | 13 / 4 | 3.26 | 2.8 | 3.30 |
| hGRP78-NDB apo | A | 2069 | 10 / 7 | >3.5 | >3.5 | >3.5 |
|  | B | 2321 | 18 / 6 | >3.5 | >3.5 | 2.74 |
| hGRP78-NDB – ADP-PO_4_ | A | 2162 | 14 / 7 | >3.5 | *2.9* | >3.5 |
|  | B | 2089 | 10 / 6 | >3.5 | *3.0* | >3.5 |
| hGRP78-NDB – ATP | A | 2096 | 12 / 7 | >3.5 | *2.80* | >3.5 |
|  | B | 2308 | 15 / 7 | >3.5 | *2.97* | 3.48 |

**Figure S1**. Scanned autoradiography of the western-blot presented in Figure 4. Lanes 2 to 8 are shown in figure 4. Molecular weight makers EZ-Run™ Prestained Rec Protein Ladder (Fisher BioReagents; lane 1), range 170 to 10 kDa (orange/red 72kDa and green 10kDa).

**L 1 2 3 4 5 6 7 8 9 10**


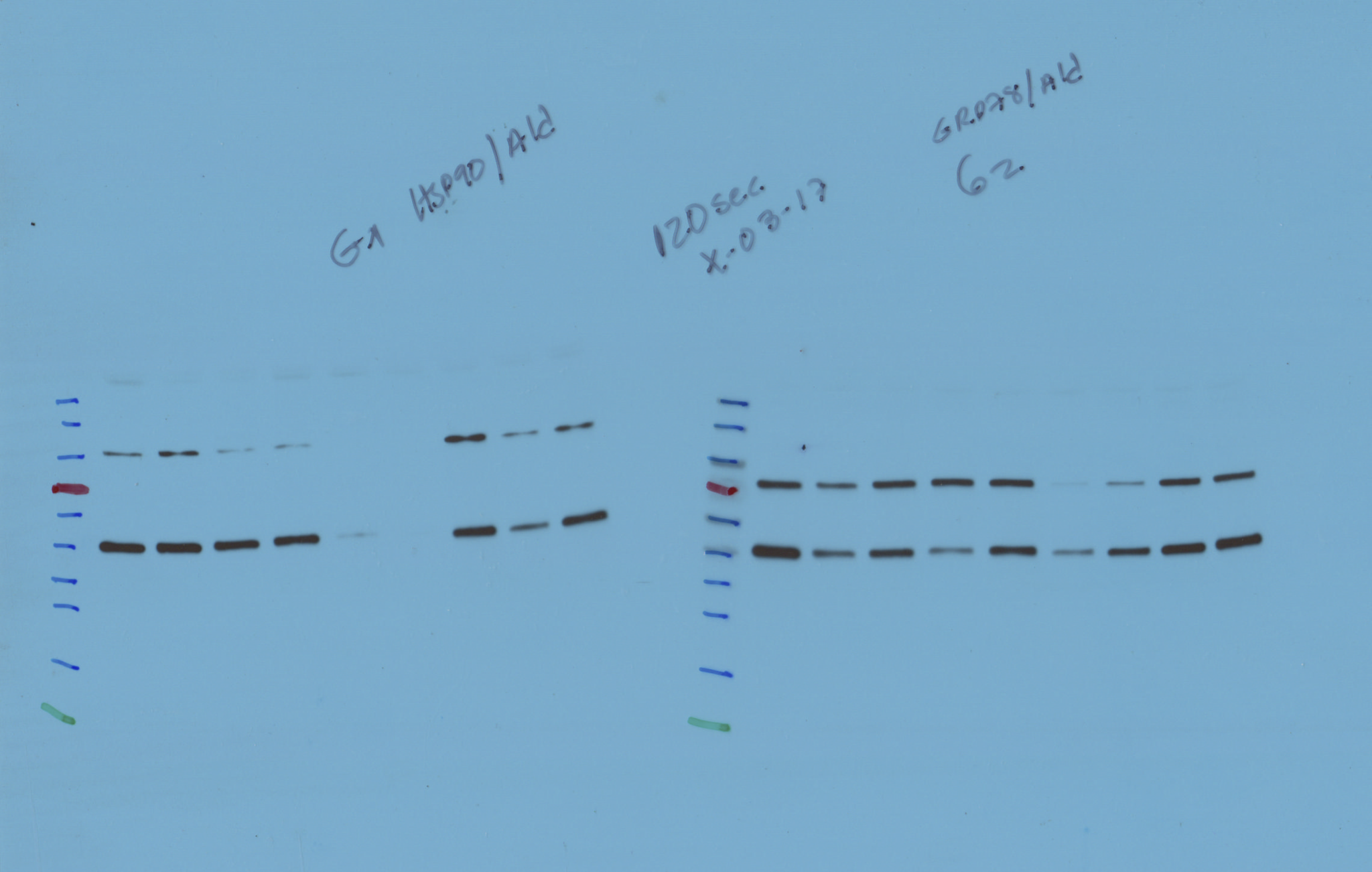

Supplement: Supplementary file 1 — Supplementary Information [file 41598_2018_28608_MOESM1_ESM.docx]
